# Development and psychometric properties of Egoism at Work Attitude Questionnaire

**DOI:** 10.3389/fpsyg.2026.1852447

**Published:** 2026-05-29

**Authors:** Marcin Wnuk

**Affiliations:** Department of Work and Organizational Psychology, Adam Mickiewicz University, Poznań, Poland

**Keywords:** confirmatory factor analysis, convergent validity, exploratory factor analysis, measure, workplace egoism

## Abstract

**Introduction:**

A selfish approach in the workplace can have harmful effects on employees and the organization. Employee egoism has received limited attention in the workplace context. Only one measure had been used to verify this phenomenon, but it was not designed for business and had some methodological limitations. This study aimed to operationalize workplace egoism and presented a measure for its verification.

**Methods:**

The construct, convergent and criterion validity, as well as internal consistency of the Egoism at Work Attitude Questionnaire (EWAQ), were examined on the three samples that consisted of Polish employees.

**Results:**

An exploratory factor analysis (EFA) involving 800 employees from Poland revealed a one-factor structure of the EWAQ, which explained 66.99% variance in the egoism attitude in the workplace. In a sample of 754 employees, confirmatory factor analysis (CFA) confirmed the good construct validity and internal consistency of EWAQ, with a Cronbach’s alpha coefficient of 0.89. These values align with Bagozzi and Heatherton’s (1994) criteria for the magnitude of factor loadings, average variance extracted, and composite reliability, which corroborate the good convergent validity of this tool. The criterion validity of the EWAQ was confirmed in two samples of Polish employees, showing correlations with dark triad traits, instrumental ethical climate, and indicators of occupational well-being.

**Discussion:**

The EWAQ is a psychometrically sound measure of egoistic attitudes at work and is recommended for use among Polish employees.

## Introduction

### Egoism – different approaches

Egoistic behaviors are a topic of significant interest in various scientific disciplines, including philosophy, psychology, medicine, sociology, management, and ethics. Egoism (from the Latin word ‘ego’) means focusing on one’s self and caring about one’s interests, as opposed to altruism (from the French term ‘altruisme’ derived from ‘autrui’ or ‘other people’ derived from the Latin word ‘alter’ or ‘other’) defined by Comte as a life to others due to a moral obligation to renounce self-interest (Comte, 1852). The phenomenon of egoism is viewed from both descriptive and normative perspectives. The descriptive approach regards this human quality as a disposition to prioritize self-interest and motivation based on one’s desires and needs, without moral judgment or indication of desirable and undesirable standards; it has its reflection in psychological egoism (Chung, 2016; Shaver, 2021). Normative egoism views egoism as a standard of human functioning that serves as a reference point for the evaluation of human behavior in societies; it designates some actions or outcomes as good, desirable, or permissible while others as bad, undesirable, or impermissible, and is divided into two types of egoism: ethical and rational (Ramanathan and Swain, 2019; Shaver, 2021). Rand (1964), a known advocate of rational egoism, stated that humans are objectively selfish and desire happiness as the highest moral purpose of their rational existence. She perceived altruism, the opposite of egoism, as irrational and leading to undesirable results, such as the limitation of self-sufficiency, avoidance of personal happiness, and compromised individual rights (Rand, 1957).

There is no possibility of considering egoism without referencing altruism as an oppositional term. Egoistic needs cannot be at the same time prosocially oriented (Batson and Shaw, 1991; Batson et al., 1995), and this dichotomy exists regardless of the fact that an egoistically motivated attitude can lead to prosocially significant benefits (Hartenian and Lilly, 2009; Omoto and Snyder, 1995). Against this dichotomy is Krebs (1991), who emphasizes the lack of differences between self-interest and the welfare of others, taking into account the genetic perspective in the case of connections between relatives (the paradox of altruism in biology) and the psychological point of view in situations where individuals perceive themselves as a unified whole.

Both of these constructs are involved within the long-term and widespread discussion regarding egoistic behaviors as only one motivational factor or the possibility of the presence of altruistic manifestation as real altruistic-oriented acting or a cover for egoistic motives (Cialdini, 1991; Maner and Gailliot, 2007). Mueller’s (1986) research has indicated two natures of human beings: a selfish nature and a cooperative-altruistic nature, which are activated depending on the context. It means that donations to charities or volunteering for community work are seemingly determined by prosocial motives, and there are other reasons for these behaviors that exist besides altruistic concerns for others’ welfare. Within social exchange (Blau, 1964), deeply grounded in human relationships, reciprocity expectation (Gouldner, 1960) strengthens altruistic motivation as a potentially beneficial and profitable endeavor for the individual.

Some scientists have tried to confirm that humans are driven not only by egoistic motives but also by intrinsically oriented altruistic behaviors. For example, Batson and Shaw (1991) and Batson et al. (1995) demonstrated that an altruistic attitude is not solely driven by selfish motives, but rather by empathy, which naturally leads to helpfulness and supportive reactions, regardless of whether they are profitable or not. Moreover, Kurt Lewin’s philosophy emphasized altruism, in addition to egoism, collectivism, and principlism, as motives behind prosocial behaviors (Batson, 2022). Batson’s approach to altruism has been criticized by representatives of sociobiology, who claim that every human action is rooted in underlying selfishness (Barash, 1982; Wilson, 1975) and can be explained by genetic egoism, emphasizing the role of genes in preserving the species. Being theoretically possible and meaningful, altruistic acts are recognized by social constructionism (Gantt and Reber, 1999) and are considered a counterpoint to biological determinism in sociobiology.

In conclusion, both normative and psychological approaches to egoism emphasize egoism as an underlying factor in human behavior. The first one emphasizes the socially oriented signposts regarding egoistic actions as permissible or impermissible and desirable or undesirable, whereas the second one glorifies egoism as an immanent and beneficial drive leading to happiness, stressing individual interests over social ones.

In this research, egoism is investigated as an attitudinal factor in the workplace, which leads to various individual, interpersonal, and organizational consequences. The primary aim is to examine whether these behaviors are beneficial or detrimental to employees and organizations. It is crucial for organizational practice and employees’ well-being because the potential implementation of changes in egoism in the workplace requires knowing whether this factor is detrimental and should be inhibited or beneficial and should be enhanced. Another perspective is that egoistic behaviors can be beneficial for employees but detrimental to an organization. To verify this, it is necessary to consider both normative and psychological perspectives on egoism.

Especially important is to show that egoism can exist on three levels: organizational, interpersonal, and individual, which are connected. Egoistic attitudes can be modified by supervisors with either selfish or altruistic leadership, as well as through the perception of an organizational ethical climate that encourages or discourages self-interest attitudes in the workplace.

### Egoism – organization and team contexts

From a business ethics perspective, it is crucial to explain how individual egoism, the perception of the environmental context at work from the perspectives of desirable and undesirable moral behaviors in the workplace (ethical climate), and leader ethics influence employees’ attitudes toward the organization, their well-being, and organizational welfare.

The ethical climate is an organizational dimension that serves as a framework to explore ethical and unethical workplace behaviors and the motivational aspects that underpin them. This phenomenon is defined as a set of shared perceptions of procedures and policies, both codified and informal, that shape expectations for ethical behavior within an organization or company (Victor and Cullen, 1987). There are five types of ethical climate: instrumental, caring, independence, rules and law, and codes. The former is geared toward self-interests on the individual level and company profit on the global level (Victor and Cullen, 1988).

Previous research has highlighted the harmful consequences of an instrumental ethical climate both on the individual and organizational levels (Friend et al., 2020; Simha and Cullen, 2012). For example, an egoistic ethical climate was negatively linked with organizational commitment (Bulutlar and Öz, 2009; Choe et al., 2017; Cullen et al., 2003; Demirtas and Akdogan, 2015; Deshpande, 1996; Kelley and Dorsch, 1991; Teresi et al., 2019), overall job satisfaction (Cullen et al., 2003; Deshpande, 1996; Wang and Hsieh, 2012), positive affect (Wang and Hsieh, 2012), promotion satisfaction (Deshpande, 1996; Tsai and Huang, 2008), co-worker and supervisor satisfaction (Deshpande, 1996; Kim and Miller, 2008), and identification with the company (Leung, 2008; Teresi et al., 2019), while it was positively associated with turnover intention (Demirtas and Akdogan, 2015), negative affect (Wang and Hsieh, 2012), deviant workplace behaviors (Peterson, 2002), and bullying behaviors (Bulutlar and Öz, 2009). It is also indirectly negatively related to organizational citizenship behavior through negative affect (Chernyak-Hai and Tziner, 2021).

Besides the ethical climate at work, which reflects an organizational perspective, the supervisor, as an interpersonal dimension in the workplace, has a significant impact on the ethical behaviors of employees. Among the different types of leadership, such as servant-like (Eva et al., 2019), paternalistic (Ötken and Cenkci, 2012), authentic, spiritual, and transformational, ethical leadership (Crews, 2015) has also been examined in the context of self-centered and selfish attitudes of managers in the workplace. Ethical leadership has been defined as “the demonstration of normatively appropriate conduct through personal actions and interpersonal relationships and the promotion of such conduct to employees through two-way communication, reinforcement, and decision-making” (Brown et al., 2005, p. 120). Ethical leadership is effected through social learning processes in which the supervisor, perceived as an organizational agent (Levinson, 1965), models expected and acceptable employee behaviors in the workplace (Brown et al., 2005). Recent research has confirmed that leader ethics constitute an antecedent of perception in the organizational climate (Neubert et al., 2009; Schminke et al., 2005). For example, in a longitudinal study, Hansen et al. (2016) found that employees’ perceptions of top management’s ethical leadership predict their views on the organizational ethical climate. In addition, Demirtas and Akdogan’s (2015) study found that ethical leadership was indirectly positively related to affective commitment and negatively correlated with turnover intention, mediated by employees’ perceived ethical climate. In the same vein, selfish and self-focused behaviors of managers are related to an egoistic organizational climate. In a sample of 258 civil servants working in various public sectors in Taiwan, authoritarian leadership positively correlated with an egoistic climate and negatively with a benevolent climate (Wu and Tsai, 2012). Ethical leadership is not only manifested indirectly through the ethical climate but also directly connected with ethical behavior (Lu and Lin, 2014). Previous research has shown that ego-driven leadership has a detrimental effect in the workplace (Kwak and Shim, 2017). Contrarily, managers’ altruism-oriented actions are linked to the benefit of employees (Sosik et al., 2009). In an exploration of the literature on the topic of egoism of employees, the author of this article found only one paper in which a supervisor’s altruistic personality was positively linked with a subordinate’s loyalty and his/her assessment of the supervisor’s benevolence (Wu et al., 2012).

The lack of measures for employees’ egoistic behaviors makes it impossible to holistically address the egoistic attitude at work, which extends beyond organizational and interpersonal perspectives. It indicates a need to define and operationalize workplace egoism as a propensity to behave in a selfish way in the workplace and to conduct preparations based on that tool to verify it. This individual perspective is presented in the subsection below.

### Egoism – individual perspective

According to egoistic motivation, which is focused on taking care of one’s welfare in psychology, two main ways to achieve well-being are recognized: hedonism and eudaimonia (Diener, 1984). Hedonism refers to a desire to maximize the intensity of pleasure and reduce pain and distress, and can be considered a selfish approach (Veenhoven, 2003). Based on Aristotle’s philosophy, eudaimonia assumes that the way to a fruitful, happy, and valuable life is living in coherence with an internal spirit called ‘dajmon,’ which reflects one’s ‘true self’ and encompasses individual talents, predispositions, values, preferences, and needs (Waterman, 1990). Research has indicated that these paths are connected to manifestations of life contentment, but only the eudaimonic approach is positively related to the well-being of relatives (Huta et al., 2012). Within positive psychology, the concept of strengths of character (Seligman, 2002), such as altruism, as antecedents of eudaimonic happiness has been developed, which explains the lack of interest in egoism and the presence in literature of a plethora of studies oriented on exploring altruistic attitudes (Corral-Verdugo et al., 2015).

There is a dearth of studies in the literature on how individual egoistic acts at work are connected to occupational well-being and attitude toward the organization, as well as the mechanisms underlying this link. Although the research on egoism is limited in volume, it has confirmed that behaviors driven by egoistic motives are associated with negative outcomes (de Vries and van Kampen, 2010). Previous studies have indicated a positive relationship between egoism and immorality (de Vries and van Kampen, 2010), Machiavellianism and psychopathy (de Vries and van Kampen, 2010; Moshagen et al., 2018; Raine and Uh, 2019), narcissism, sadism, impulsivity, aggression, spitefulness (Moshagen et al., 2018), pretentiousness (de Vries and van Kampen, 2010), neuroticism (de Vries et al., 2009; de Vries and van Kampen, 2010; Weigel et al., 1999), hostile sexism in men (Schrödter et al., 2021), and tolerance of sexual harassment and victim blaming (Weigel et al., 1999). Additionally, egoism is negatively related to positive personality traits, such as honesty–humility (de Vries et al., 2009; de Vries and van Kampen, 2010), extraversion (de Vries et al., 2009; Weigel et al., 1999), openness to experience (de Vries and van Kampen, 2010; Weigel et al., 1999), agreeableness (de Vries et al., 2009; Weigel et al., 1999), and conscientiousness (Weigel et al., 1999). Selfishness is also positively related to antisocial and histrionic personality, anxiety, and depression (Raine and Uh, 2019), and negatively correlated with altruism, affective empathy, warmth (Raine and Uh, 2019), as well as the light triad of personality, such as Humanism, Kantianism, and faith in humanity (Kaufman et al., 2019).

Notably, in all of the above research, the Egoism Scale (Weigel et al., 1999) was used to evaluate egoistic behaviors. The cited scale verifies egoism by emphasizing dismissive attitudes toward obeying social norms, rules, codified laws, and non-articulated standards of social coexistence, due to the desire for pleasure, advantage, or well-being. This tool has some methodological shortcomings and limitations regarding its internal and convergent validity.

Authors declared that 32 of the original 58 items loaded on a single factor at 0.21 or above, which is not an acceptable level because the standard is 0.4 or even 0.6 in a more restrictive approach (Henson and Roberts, 2006). They did not present the percentage of variance explained by those items. An item’s minimum value-loaded single factor is not the reason to reduce the items to 20, but the average inter-item correlation and Cronbach’s alpha coefficient. It was not revealed whether all single items loaded this factor more than 0.4, as an established minimum, or not, and whether the one-factor solution was well-fitted to the data. The authors also claimed that the one-factor model was not a perfect fit to the data, but it was preferred over multifactor solutions due to its parsimony. Using item-scale correlations as a criterion to assess convergent validity has shown that this measure has weak convergent validity because, in two research samples, five of 20 items in the Egoism Scale correlated with this measure below the threshold value of 0.3 (Nurosis, 1994).

The absence of a measure to examine egoism from the perspective of individual disposition, which drives someone to behave egoistically, the weak internal and convergent validities of the available measure, and the potential difficulties in adapting it to the business domain were some of the reasons behind the work conducted on the new tool. The main purpose of the author in creating a new measure to verify individual egoism, complementary to the organizational context (egoistic ethical climate) and interpersonal approach (relationship with an unethical supervisor), was to capture egoistic behaviors at work from a broader perspective than previously adopted.

Taking into account Lewin’s (1951) theory, at work, a person is motivated by their individual needs, desires, goals, and values. From both organizational and interpersonal perspectives, his behavior is shaped by expectations set by the organization (ethical climate), his supervisor (ethical leadership), and his coworkers. These factors are interrelated, meaning that individual employee motivation is influenced and adjusted to meet the expectations of supervisors, coworkers, and the organization. This means that egoistic attitudes at work will be influenced by the ethical climate, leaders, and team ethics. Depending on these factors, egoistic behaviors and attitudes will be either strengthened and rewarded or punished and stigmatized (Babin et al., 2000). Adopting a holistic perspective is a highly effective strategy for comprehensively addressing egoistic behaviors at work and creating a reliable and accurate picture of this phenomenon.

The relationship between egoistic attitude and well-being still remains a “terra incognita,” which requires extensive exploration. Preparing a new tool to verify egoism at work (workplace egoism) is also necessary to determine whether this attitude is associated with beneficial or harmful outcomes. Focusing on one’s own welfare, pleasures, needs, and desires (Baier, 1990; Chung, 2016; Lemos, 1960), even at the expense of others’ needs and goals, should lead to achieved satisfaction and well-being. On the other hand, hyper-intention in pursuit of happiness can have the opposite effect, leading to existential frustration caused by a lack of meaning and purpose in life (Frankl, 1992). It is essential to determine whether an egoistic attitude is worthwhile to develop and strengthen, or whether it should be inhibited and limited as a factor that harms individual well-being and organizations. Based on these results, it can serve to prepare intervention and therapeutic programs centered on changing the level of employee egoism in a desirable direction.

## Methods

### Egoism at work attitude questionnaire (EWAQ) preparation

Preliminary 25 items regarding egoistic attitude at work were based on the definition of egoism as excessive concern with self-interest and welfare at the expense of others. An egoistic attitude prioritizes self-care and disregards the rights of others when these others interfere with one’s pursuit of economic and personal gain (Weigel et al., 1999). It means that egoistic behavior can be positive for both coworkers and employers, but only if it aligns with the needs and desires of egoistic-oriented employees. It was assumed that egoistic attitudes at work should be measured without considering whether these behaviors are morally accepted and beneficial to society, or rationally oriented, independent of the postulates of ethical or rational egoism (Ramanathan and Swain, 2019; Shaver, 2021).

To avoid the potential influence of egotism on egoism as a similar and overlapping construct, only the most substantial manifestations of egoistic attitudes were taken into account. Egotism is a drive to maintain and develop a positive self-image, which, similar to egoism, is negatively correlated with humility; however, unlike egoism, it is not connected with emotionality and is positively related to extraversion (de Vries et al., 2009).

Following Herman’s (1992) proposition, three aspects of egoistic attitudes were established on the dimensions: selfish–not selfish, instrumental approach to obeying rules and values, non-instrumental approach to obeying rules and values, and self-interest against the interests of workmates and employer – lack of self-interest against the interests of workmates and employer.

The preliminary 25-question survey regarding the above aspects of egoistic attitudes, with responses on a five-point Likert Scale from ‘completely disagree’ to ‘completely agree’, was prepared by the author of this article and then translated from Polish to English and then from English to Polish by applying a backward translation solution. The created items were assessed for internal validity by three competent psychologists using three previously established criteria for workplace egoism: selfish–not selfish; instrumental approach to obeying rules and values; and self-interest against the interests of workmates and the employer. Twenty items were selected for further analysis, which, according to all three competent psychologists, met the above criteria for workplace egoism and served as good indicators of this phenomenon.

### Statistical analysis

Statistical analyses were conducted using IBM SPSS version 26.0 (IBM Corp., 2019) and AMOS version 26.0 (Arbuckle, 2019). AMOS was applied only for confirmatory factor analysis (CFA). Due to potential methodological problems, such as the danger of overfitting, exploratory factor analysis (EFA) and CFA were examined in the two different samples (Fokkema and Greiff, 2017). According to the standard, EFA was used to establish the factorial structure of the newly prepared measure, and CFA was used to confirm this structure in the other sample (Hurley et al., 1997). Construct validity was tested in Sample 1 using EFA and in Sample 2 using CFA. Internal consistency and convergent validity were computed in Sample 2. Additionally, criterion validity was calculated in samples 2 and 3. The goodness-of-fit indices for the CFA model, along with acceptable thresholds for each, are presented in Table 1. Samples 1 and 3 were recruited through a professional research panel, but, due to financial limitations, participants in Sample 2 were selected using the snowball method.

**Table 1:** Set of fit-to-model indicators with acceptable threshold levels.

Fit indicators	Acceptable threshold levels
Chi-square *χ*^2^	Low *χ*^2^ relative to degrees of freedom with an insignificant *p*-value (*p* > 0.05)
Relative *χ*^2^ (*χ*^2^/df)	3:1 (Kline, 2005)
RMSEA	Values less than 0.07 (Steiger, 2007)
GFI	Values greater than 0.95
AGFI	Values greater than 0.95
SRMR	Values less than 0.08 (Hu and Bentler, 1999)
NFI	Values greater than 0.95 (Hu and Bentler, 1999)
CFI	Values greater than 0.95 (Hu and Bentler, 1999)

### Construct validity

#### Sample 1

The study comprised 800 participants with employment contracts in organizations of varying sizes and across different business sectors. The data were collected by an organization specializing in this kind of project through the Manulo research panel. All participants provided written online consent to take part in the research. They were advised of the anonymity of their responses and could withdraw from the study at any time without consequences. The criteria for including research were adulthood, Polish citizenship, at least 1 year of seniority in their current jobs, and employment in Poland.

Participants were informed that the research was anonymous and voluntary. Every individual agreed to participate in the study. The sample comprised 54.4% women and 45.6% men, with a mean age of 43.79 years (SD = 12.66). Six (0.7%) participants had an elementary education, five (0.6%) had lower secondary education, 28 (3.5%) had vocational education, 316 (39.5%) had a secondary education, 435 (54.4%) had completed higher education, and 10 (1.3%) had a doctoral degree. Regarding their positions, 574 (71.8%) were ordinary workers, 127 (15.9%) were low-level managers, 78 (9.8%) were mid-level managers, and 21 (2.6%) were senior-level managers. The average seniority of the research participants in the present work was 11.23 (SD = 10.32).

#### EFA

The EFA was conducted on 20 selected items. The sample size was enough to meet EFA standards, taking into account the suggested closeness of items with the construct (MacCallum et al., 1999), a minimal arbitrary number of participants (100–200), and the number of research participants per item (Bryman and Cramer, 1997; Hair et al., 2010). The values of skewness (less than 2.0) and kurtosis (less than 7.0) (see Table 2) (Curran et al., 1996) imply a data distribution close to the normal distribution, which is required in EFA. The Kaiser–Meyer–Olkin sampling adequacy test value yielded 0.934, which is higher than the acceptable minimum of 0.7. Bartlett’s sphericity test resulted in 4623.170, df = 28, and *p* < 0.01, which confirmed the adequacy of the research sample.

**Table 2:** Descriptive statistics (*n* = 800).

Number of items	Mean	Standard deviation	Skewness	Kurtosis	Cronbach’s alpha if deleted item
1	2.41	1.06	0.44	−0.49	0.92
2	2.49	1.09	0.32	−0.68	0.92
3	2.22	1.12	0.54	−0.66	0.91
4	2.29	1.12	0.45	−0.75	0.92
5	2.89	1.13	−0.05	−0.75	0.93
6	2.26	1.12	0.49	−0.67	0.91
7	2.28	1.07	0.41	−0.70	0.92
8	2.22	1.10	0.62	−0.41	0.91

To determine the number of factors, two methods were applied: Cattell’s scree test and Kaiser–Guttman’s ‘eigenvalues are greater than one’ criterion (Guttman, 1954; Kaiser, 1960). The employed methods confirmed the one-factor solution. The eigenvalue was more than one only for one factor (5.35).

A 0.6 value cut-off ratio of factor loading by the items was used (Henson and Roberts, 2006; Park et al., 2002). All 8 selected items loaded a factor of more than 0.6, which was acceptable (Table 3). The extracted one-factor solution explained 66.99% of the variance in the egoism attitudes in the workplace construct, which exceeded the 50% required standard (Pallant, 2016). Internal consistency measured by Cronbach alpha coefficient was 0.93, which was a satisfactory result.

**Table 3:** EFA results of EWAQ (*n* = 754).

Items	Factor loadings
At work, only my business matters [W pracy liczy się tylko mój interes]	0.82
At work, you should only take care of yourself [W pracy trzeba dbać tylko o siebie]	0.74
At work, I reckon only with those who can help me further my career [W pracy liczę się tylko z tymi ludźmi, którzy mogą pomóc mi w dalszej karierze zawodowej]	0.77
To be successful at work, you should bend moral rules [Aby osiągnąć sukces w pracy trzeba naginać zasady moralne]	0.75
I am loyal to the organization only when it benefits me [Jestem lojalny wobec organizacji tylko wtedy, gdy przynosi mi to korzyści]	0.79
It does not bother me that the development of my professional career can take place at the expense of my workmates [Nie przeszkadza mi to, że rozwój mojej kariery zawodowej może się odbyć kosztem moich kolegów i koleżanek z pracy]	0.76
I do not obey the company rules if it is not in my interest [Nie przestrzegam zasad panujących w firmie w organizacji/firmie, jeśli nie jest to zgodne z moim interesem]	0.7
The most important thing is my well-being at work. I do not care about the rest of the employees [Najważniejsze, abym to ja miał się dobrze w pracy, reszta pracowników mnie nie obchodzi]	0.77

#### Sample 2

The study involved 754 full-time employees with employment contracts across various companies and businesses from different sectors. The criteria for including research were adulthood, possessing a contract of employment, and employment in Poland. Participants were reached through snowball sampling via social media, such as LinkedIn. Members of the LinkedIn society were encouraged to fill out the online questionnaire and were recommended to do so to their friends.

All participants provided written online consent to participate in the study. They were advised of the anonymity of their responses and had the option to withdraw from the study at any time without consequences. The sample consisted of 450 (59.6%) women and 304 (40.4%) men. The mean age of the participants was 29.54 years (SD = 10.22), and the mean of seniority was 8.19 (SM = 9.17). Four attained elementary education (0.5%), 29 attained vocational education (3.8%), 312 attained secondary education (41.4%), and 409 attained a higher level of education (34.5%). For the level of position held, there were 260 ordinary workers (34.5%), 319 independent specialists (24.3%), 57 low-level managers (7.6%), 86 middle-level managers (11.4%), and 32 senior-level managers (4.2%).

#### CFA

The CFA for the one-factor structure of the egoism attitude in the workplace questionnaire was conducted. The achieved results (RMSEA = 0.000 [0.000, 0.029], SRMR = 0.007, NFI = 0.99, GFI = 0.99, CFI = 1, statistics Chi^2^ (Chi^2^ statistic value – CMIN) = 7.9, df = 11, *p* = 0.722, CMIN/DF = 0.71, and PCLOSE = 0.99) confirmed the good fitness to the data.

Furthermore, the potential gender differences in egoism at work were verified. Results showed that women and men differ in their egoistic attitudes at work [*t*(754) = −5.15, *p* < 0.01] and that women (*M* = 15.73; SD = 5.15) are less egoistic at work than men (*M* = 18.02, SD = 6.62).

##### Internal consistency

To examine internal consistency, the Cronbach’s alpha coefficient and inter-item correlations were used. All of these methods confirmed the good internal validity of this measure. The Cronbach alpha coefficient value was 0.89, which can be interpreted as a high value, especially for developing a questionnaire (Bowling, 1997). Furthermore, the outcomes of the inter-item correlations were found to be within the required (acceptable) range of results, between < 0.3 and> 0.7 (Ferketich, 1991) (see Table 4). This means that the items are good indicators of a measurable construct that reflects the wide range of its research aspects, but they differ from each other sufficiently, being separate items (Kline, 2005).

**Table 4:** Correlation matrix for Sample 2 (*n* = 754).

	Item 1	Item 2	Item 3	Item 4	Item 5	Item 6	Item 7
Item 2	0.69**						
Item 3	0.46**	0.55**					
Item 4	0.41**	0.44**	0.50**				
Item 5	0.53**	0.56**	0.57**	0.53**			
Item 6	0.52**	0.56**	0.59**	0.57**	0.58**		
Item 7	0.44**	0.44**	0.51**	0.51**	0.53**	0.53**	
Item 8	0.53**	0.64**	0.61**	0.54**	0.57**	0.66**	0.51**

**p < 0.01.

##### Convergent validity

Convergent validity was tested following Bagozzi and Heatherton’s (1994) criteria: the magnitude of most factor loadings (threshold 0.50), average variance extracted (threshold 0.50), and composite reliability (qC) (threshold 0.60–0.70). According to the results presented in Table 5, the values of factor loading were higher than the established standard (from 0.64 to 0.84), the average variance extracted was more than 50%, and the composite reliability was much higher than 0.7 (Byrne, 2013). These results demonstrate that the EWAQ exhibits good convergent validity.

**Table 5:** Results of methods used to verify convergent and divergent validities and MAP (*n* = 754).

Item	Item-scale correlations	Latent variables	Standardized loadings	Square of standardized loadings	Sum of square of standardized loadings	Number of indicators	Average variances extracted (AVE)	Composite reliability
1	0.65	Egoism	0.64	40.96	439.01	8	54.87	0.91
2	0.71		0.75	56.85				
3	0.70		0.73	53.72				
4	0.64		0.71	50.27				
5	0.71		0.75	56.10				
6	0.74		0.79	62.73				
7	0.63		0.69	48.16				
8	0.76		0.84	70.22				

##### Criterion validity

To assess criterion validity, the EWAQ was correlated with positive and negative indicators of occupational well-being and with attitude toward the organization. It was expected that workplace egoism would be correlated with occupational well-being indicators. The following hypotheses were tested:

*Hypothesis 1*: Workplace egoism is negatively correlated with job satisfaction, meaning in work, and organizational gratefulness.

*Hypothesis 2*: Workplace egoism is positively correlated with job stress.

##### Measures

Job stress was assessed using a short version of the Polish adaptation of the Perceived Stress at Work Scale (Chirkowska-Smolak and Grobelny, 2016; Cohen and Janicki-Deverts, 2012), which consisted of five of the 10 original items from the original version. Participants responded on a 5-point Likert scale ranging from 5 = never to 1 = almost always (Cohen and Janicki-Deverts, 2012). The good psychometric properties of this version, as measured in Polish conditions, were confirmed (Wnuk, 2018, 2019). One example item from this instrument is “In the last month, how often have you been upset because of something that happened unexpectedly?”

To measure meaning in work, the Work and Meaning Inventory (WAMI; Steger et al., 2012) was used. The original three-dimensional structure of the WAMI was not replicated for Polish conditions, as the two-factor solution is a good fit for the data (Puchalska-Kamińska et al., 2019). The personal meaning dimension was used, which consists of six items rated on a five-point Likert scale, ranging from (1) absolutely untrue to (5) absolutely true. One example item from this instrument is “I have a good sense of what makes my job meaningful”.

Job satisfaction was measured using one question, “How satisfied are you generally with work?” Participants had to respond using a 10-point scale, ranging from 1 (very dissatisfied) to 10 (very satisfied).

The Gratitude Toward the Organization Scale (Wnuk, 2020) consists of two factors: gratitude as a moral norm and gratitude as a commitment to reciprocity. Each question was rated on a 5-point Likert scale, ranging from 1 (strongly disagree) to 5 (strongly agree). This measure has good psychometric properties. In this study, items regarding gratitude as a commitment to reciprocity were used. One example item from this instrument is “I have received a lot while working here”.

##### Results

The results of the r-Person correlation coefficients for EWAQ and the measures used as criterion validity, and the alpha Cronbach’s coefficients for these tools, are presented in Table 6. In line with Hypothesis 1, a negative relationship was noted between workplace egoism and job satisfaction, meaning in work, and gratitude toward the organization. Also, Hypothesis 2, which posited a negative relationship between workplace egoism and job stress, was supported. It was found that workplace egoism was positively related to job stress. Additionally, older and better-educated employees had a lower level of egoism than their younger and less educated counterparts.

**Table 6:** Correlation coefficient results between the EWAQ and measures used for criterion validity (*n* = 754).

Measures	Cronbach’s alpha coefficient	EWAQ
Job satisfaction	–	−0.20**
Meaning in work	0.92	−0.34**
Job stress	0.85	0.13**
Gratitude toward the organization	0.9	−0.28**
Level of the position held	–	0.04
Overall seniority	–	−0.05
Education	–	−0.14**
Age	–	−0.08*

*p < 0.05, **p < 0.01.

#### Sample 3

This study involved 953 full-time employees, possessing Polish citizenship with employment contracts and at least 1 year of seniority in their current jobs. The data were collected by a company specializing in collecting data through the Manulo research panel. All participants expressed their written informed consent to participate in the study. They were informed about the anonymity of their responses and the possibility of withdrawing from the study at any time without consequences. The sample was balanced in terms of gender and education, comprising 522 women (54.8%) and 431 men (45.2%). The mean age of the participants was 44.14 years (*SD* = 11.06), with an average seniority in the present work of 11.92 (SD = 9.67). Two (0.2%) participants had an elementary education, six (0.6%) had lower secondary education, 80 (8.4%) had vocational education, 327 (34.3%) had a secondary education, and 538 (56.5%) had completed higher education. Regarding their positions, 618 (64.8%) were ordinary workers, 173 (18.2%) were low-level managers, 117 (12.3%) were mid-level managers, and 45 (4.7%) were senior-level managers.

##### Criterion validity

Due to some overlaps with workplace egoism on the one hand but differences on the other hand, the following constructs were employed to evaluate criterion validity for the EWAQ: dark triad traits, instrumental ethical climate, counterproductive organizational behaviors, and altruism. The following hypotheses were examined:

*Hypothesis 1*: Workplace egoism is positively related to dark triad traits, such as narcissism, Machiavellianism, and psychopathy.

*Hypothesis 2*: Workplace egoism is positively related to instrumental ethical climate.

*Hypothesis 3*: Workplace egoism is positively related to counterproductive organizational behaviors.

*Hypothesis 4*: Workplace egoism is negatively related to altruism.

Given the similarity between concepts used to verify criterion validity and egoism at work, I expected that they would correlate at least moderately, but not strongly, with egoism at work. To assess the correlation magnitude between egoism in the workplace and the measures used to verify the criterion validity, the Guilford and Fruchter (1978) standard was adopted, according to which the moderate correlation range from 0.3 to 0.7.

##### Measures

The dark triad was verified through the Polish version of the Dirty Dozen Scale (Czarna et al., 2016). The three-dimensional internal structure of this measure was confirmed. Each dimension (narcissism, Machiavellianism, and psychopathy) is reflected by four indicators, answered on a 5-point Likert scale (from “strongly agree” = 5 to “strongly disagree” = 1). Good internal consistency and test–retest reliability of this tool were confirmed in the Polish population (Czarna et al., 2016). One example item from this instrument in reference to Machiavellianism is “I tend to manipulate others to get my way”, regarding narcissism, “I tend to want others to admire me”, and regarding psychopathy, “I tend to lack remorse”.

The instrumental work climate was examined by the items from the Polish version of the Ethical Work Climate Questionnaire (EWCQ) (Wnuk et al., 2025), which are indicators of an instrumental type of ethical climate. This tool verifies employees’ perceptions of an ethical work climate and categorizes it into the following types: instrumental, caring, law and code, rules, and independence (Victor and Cullen, 1988; Wnuk et al., 2025). The five-factor structure of the EWCQ was reflected in the study’s outcomes, as evidenced by the good reliability measured by Cronbach’s alpha coefficient. One example item from this instrument is “In this company, people protect their own interests above all else”.

Altruism was measured using the organizational citizenship behaviors scale developed by Podsakoff et al. (1990). This tool has a five-dimensional structure (conscientiousness, sportmanship, civil virtue, courtezy and altruism) and contains 24 items. Five of them regard altruism. The participants responded on a 7-point Likert scale (from “strongly agree” = 7 to “strongly disagree” = 1). One example item from this instrument is “Helps others who have been absent”.

Counterproductive work behaviors were assessed using the Polish version of the Counterproductive Work Behavior – Checklist (CWB-C) (Baka and Derbis, 2015). This tool consists of two categories of counterproductive work behavior, such as CWB-O (counterproductive work behavior – organization) and CWB-P (counterproductive work behavior – people/interpersonal). CWB-C has satisfactory psychometric properties, as evidenced by internal consistency and internal and criterion validity (Baka and Derbis, 2015). Among counterproductive work behaviors directed against the organization, such as theft, sabotage, withdrawal, or production deviance, the items regarding withdrawal were chosen in this study. One example item from this instrument is “Taken a longer break than you were allowed to take”.

##### Results

The results of r-Pearson correlation coefficients are presented in Table 7. This table also contains the alpha Cronbach’s coefficients regarding measures used as criterion validity. The alpha Cronbach’s coefficient of EWAQ in this study was 0.92.

**Table 7:** Correlation coefficient results between the EWAQ and measures used for criterion validity (*n* = 953).

Measures	Cronbach’s alpha coefficient	EWAQ
Narcissism	0.89	0.52**
Machiavellianism	0.91	0.63**
Psychopathy	0.85	0.61**
Instrumental ethical climate	0.72	0.44**
Counterproductive work behaviors	0.88	0 54**
Altruism	0.93	−0.43**

**p < 0.01.

Every four hypotheses were confirmed. Workplace egoism was moderately positively correlated with narcissism, Machiavellianism, psychopathy (H1: supported), instrumental ethical climate (H2: supported), and counterproductive organizational behaviors (H3: supported), and was moderately, negatively related to altruism in the workplace (H4: supported).

## Discussion

The purpose of this study was to operationalize egoism at work and to psychometrically test the EWAQ as a tool for measuring it. According to the psychological egoism approach, egoism determines a human being’s activities, focusing on achieving well-being and prioritizing self-interest (Shaver, 2021). This topic has been neglected in the literature, and the present research aimed to draw attention to egoistic behaviors at work as a significant phenomenon in business ethics, complementing the instrumental ethical climate on the organizational level.

Egoism is an antisocial behavior reflected in attitude toward one’s work, employer, and co-workers. It consists of caring only for one’s interest, an instrumental treatment of principles, rules, and values by observing them only when they give tangible benefits, and the belief that one’s interest does not go hand in hand with the interests of other employees, which, at best, leads to indifference to their needs, desires, and aspirations. It assumes that the employees undertake only actions that favor their well-being, without the possibility of taking actions aimed at the good of others, such as any altruistic or helpful and supportive behavior.

A psychometric evaluation of the EWAQ, a newly developed tool to measure workplace egoism, was conducted, confirming its statistical soundness. As demonstrated, EWAQ has acceptable internal validity and encompasses items that are appropriate indicators of an egoistic attitude at work. The one-factor structure of this measure was confirmed by CFA. It was demonstrated that EWAQ exhibits good internal consistency, as confirmed by the Cronbach’s alpha coefficient and inter-item correlations. The magnitude of factor loadings, average variance extracted, and composite reliability techniques clearly indicated that convergent validity was satisfied.

Moreover, the criterion validity of the EWAQ was confirmed by its correlation with measures of occupational well-being and attitude toward the organization, showing that workplace egoism is an undesirable phenomenon that should be limited to ensure employees’ flourishing. It also aligns with previous research, the egoistic behaviors in the workplace were positively related to Machiavellianism and psychopathy (de Vries and van Kampen, 2010; Moshagen et al., 2018; Raine and Uh, 2019; Wnuk, 2025), and narcissism (Moshagen et al., 2018; Raine and Uh, 2019; Wnuk, 2025), contributing in discussion about egoism as a core of dark triad traits (Moshagen et al., 2018). Also, a positive, moderate relationship between workplace egoism and perceptions of an instrumental ethical climate and counterproductive work behaviors, and a negative relationship with workplace altruism, confirmed the criterion validity of the EWAQ. What is important to note is that, as it was found, on the organizational level, an instrumental ethical climate can favor an egoistic attitude at work on the individual level, providing incentives for expressions of behaviors directed at self-centered needs and values, such as a desire for power, exploitation of others, or competition, at the expense of the well-being of other employees.

The EWAQ should be further developed and adapted for use in other social contexts that differ from those in Poland. It is essential to verify whether, as this study states, gender differences are not influenced by cultural factors. In Weigel et al. (1999), women were found to be less egoistic than men, suggesting that gender differences are universal and independent of cultural influence. On the other hand, these gender differences seem to be a more complex phenomenon that requires further investigation. It is possible that, similar to altruism, gender differences in egoistic behaviors are conditioned by other non-cultural factors. Previous studies have indicated a more altruistic attitude of women in comparison to men (Kamas et al., 2008). However, others have suggested that it is connected to the closeness of the person being the target of altruistic behavior (Decker et al., 2008), gender-biased expectations of altruism (Salgado, 2018), or the level of empathy (Kashirskaya, 2020).

In conclusion, EWAQ is a tool with good psychometric properties, designed to verify workplace egoism, and is recommended for use in organizations that employ Polish workers. Adapting this tool to different countries, especially those culturally diverse from Poland, should be the next step in its development.

### Limitations and future research

This research has some limitations. The generalizability of the research outcomes is limited to Polish employees, who are, in the vast majority, representatives of the Roman Catholic Church (Pew Research Center, 2018). Besides the religious homogeneity, also in reference to religious practices, Poles are very engaged in this kind of devotion (Pew Research Center, 2018), which can influence their egoistic attitude in the workplace. Future research should be conducted in more religiously and culturally diverse settings, in more secular nations, and among representatives of religious affiliations other than Roman Catholic.

The study participants in Sample 2, compared with those in Samples 1 and 3 (recruited through a professional research panel), were recruited through the snowball method, which is generally less rigorous and more prone to selection bias, potentially affecting the research’s generalizability. EWAQ, as with measures used to verify constructs that examine criterion validity, such as dark triad traits or counterproductive organizational behaviors, can be prone to social desirability bias, especially when assessed through self-report. Workplace egoism is an unwanted attitude, so it is possible that research participants would respond to EWAQ questions in a particular way to present themselves in a better light. To limit social desirability bias, the future study should consider collecting data on workplace egoism from other sources, such as supervisors or workmates, and compare the results with self-descriptive evaluations of a particular employee.

Further exploration of the EWAQ is needed, especially within prosocial personality traits, such as the light triad (Humanism, Kantianism, and faith in humanity). This study, like previous research, indicates that dark triad personality traits are positively related to selfishness, whereas light triad traits are negatively correlated with this antisocial behavior (Kaufman et al., 2019). It is essential to examine the role of ethical leadership and ethical organizational climate in underpinning the relationship between workplace egoism and occupational well-being, as well as attitudes toward the organization. Based on the person-organization fit concept (Kristof, 1996), verification of whether instrumental ethical climate and egoistic leadership with employees’ egoism in an interactive way positively predict work satisfaction could be an interesting and potentially fruitful area of investigation. According to trait activation theory (Tett and Burnett, 2003), egoistic behavior in the workplace should have a place in an organizational environment that will encourage this kind of attitude, especially among employees with latent potential to express egoistic aspirations, desires, and needs, such as employees with dark triad traits. This phenomenon can be strengthened or weakened by cues and incentives from supervisors, who serve as personification of the organization (Levinson, 1965), depending on their egoistic or altruistic leadership style. This study observed a coherence between the instrumental ethical climate and workplace egoism, aligning with the expected work attitude, and demonstrated that self-oriented behaviors are desirable in this type of work ethical environment.

This study suggests that, aside from instrumental ethical climate, an egoistic attitude at work may be a detrimental factor for employees’ well-being and their attitude toward the organization; however, a further in-depth investigation using the EWAQ is needed. It is recommended that future studies should focus on factors preventing workplace egoism, taking into account the opposite of instrumental ethical climate type, like a caring climate (Simha and Cullen, 2012), aimed at the well-being of every employee through cooperation, empathy, and compassion, not rivalry, cynicism, and conviction that the ends justify the means. On the interpersonal level, a promising area for further investigation is to explore how altruistic leadership can mitigate subordinates’ egoistic attitudes, especially when they tend to exhibit such behavior.

## Data Availability

The raw data supporting the conclusions of this article will be made available by the authors, without undue reservation.
